# A comparison of the TempO-Seq and Affymetrix microarray platform using RTqPCR validation

**DOI:** 10.1186/s12864-024-10586-7

**Published:** 2024-07-03

**Authors:** Matthias M. Wehr, Stella Marie Reamon-Buettner, Detlef Ritter, Jan Knebel, Monika Niehof, Sylvia E. Escher

**Affiliations:** Fraunhofer Institute for Toxicology and Experimental Medicine, Nikolai-Fuchs-Str. 1, 30625 Hannover, Germany

**Keywords:** TempOSeq, Affymetrix, Microarray, RTqPCR, RT2 profiling arrays, Chemical perturbation, Transcriptomics

## Abstract

Next-generation risk assessment relies on mechanistic data from new approach methods, including transcriptome data. Various technologies, such as high-throughput targeted sequencing methods and microarray technologies based on hybridization with complementary probes, are used to determine differentially expressed genes (DEGs). The integration of data from different technologies requires a good understanding of the differences arising from the use of various technologies.

To better understand the differences between the TempO-Seq platform and Affymetrix chip technology, whole-genome data for the volatile compound dimethylamine were compared. Selected DEGs were also confirmed using RTqPCR validation. Although the overlap of DEGs between TempO-Seq and Affymetrix was no higher than 37%, a comparison of the gene regulation in terms of log2fold changes revealed a very high concordance. RTqPCR confirmed the majority of DEGs from either platform in the examined dataset. Only a few conflicts were found (11%), while 22% were not confirmed, and 3% were not detected.

Despite the observed differences between the two platforms, both can be validated using RTqPCR. Here we highlight some of the differences between the two platforms and discuss their applications in toxicology.

**Supplementary Information:**

The online version contains supplementary material available at 10.1186/s12864-024-10586-7.

## Introduction

In recent decades, technologies that analyze changes in the transcriptome, have advanced significantly. Several methods are now available, including microarrays as well as next generation sequencing methods [1]. Transcriptome data can reveal early changes at the level of intracellular processes in both in vitro and in vivo assays and are, therefore, seen as a promising tool for next generation risk assessment (NGRA). NGRA relies on human-centric models to gain mechanistic insights into the development of adverse outcomes relevant to human safety assessment. To integrate such data into hazard and later risk assessment, it is essential to have a good understanding of the advantages and limitations of different technologies.

Chip technologies use probes to measure the hybridization of fluorescent markers - placed on arrays. These arrays can be customized, but manufacturers such as Affymetrix and Agilent, provide common layouts that cover the whole transcriptome of a species. The Clariom D Array from Affymetrix is available for use on human, rat, and mouse samples. It covers many non-coding and small transcripts, in addition to the coding transcriptome. Chip technologies require RNA purification as a first step, and are, therefore, more time and cost- intensive compared to recently developed targeted next- generation sequencing technologies (see next paragraph). However, they have proven to be robust and reproducible, and are thus widely used to identify biomarkers and transcriptional signatures for toxicological endpoints. For example, they have recently been used for the identification of toxicological signatures of developmental toxicants [2].

Meanwhile, next generation RNA-sequencing (NGS) methods are based on sequencing where labeled nucleotides provide a base-by-base readout. Several platforms, such as Illumina and Ion Torrent (Thermo Fischer), scale this process by parallelization. NGS techniques include library preparation, cluster formation, sequencing, and alignment. In contrast, Templated Oligo-Sequencing (TempO-Seq) employs a „hybridization to detector oligo“ - step that simplifies library preparation. This technique has the advantage that only targeted sequencing is performed, thus eliminating the need for RNA isolation. This adjustment not only reduces costs, but also resolves difficulties in reading depth [3]. The targeted techniques TempO-Seq and Affymetrix share the commonality that they are based on a pre-designed panel of oligonucleotides which target specific sequences. TempO-Seq aims to solve difficulties of hybridization specificity with a requirement of perfect alignment, of detector oligos to highly specific target sequences, before ligation [3]. In contrast, Affymetrix employs mismatch and control probes to allow for subtraction of noise and cross-hybridization computationally [4].

NGS methods allow high- throughput testing of samples and have, therefore, gained significant importance in screening of compounds to investigate gene expression changes in a time and concentration -dependent manner. Examples of this context include the ToxCast and Tox21 projects, which recently published a large inventory of omics data from 120 in vitro and in vivo assays [5–7]. Dose- response data are of particular interest because these data allow for the derivation of benchmark concentrations that describe the onset of gene deregulation and pathway perturbation. In the near future, such data may be used for in vitro to in vivo extrapolation to set a point of departure for risk assessment [8].

Several studies have investigated the concordance between chip and next- generation sequencing technologies [9–11]. These studies showed consistencies between TempO-Seq analysis and other more established techniques [9]. RNA-Seq has both advantages such as higher statistical power, and disadvantages, including more noise and expense. Two studies reported an overlap of about 80 to 90% differentially expressed genes (DEGs) for RNA-Seq and microarray techniques [11, 12].

Reverse- transcription quantitative PCR (RTqPCR) is considered the gold standard for measuring mRNA expression in both in vitro and in vivo studies. RTqPCR is a widely established technique for detecting mRNA expression. It enables the quantification of gene expression changes with a high dynamic range and high sensitivity, and depending on probe design, high specificity. These properties make this technique applicable for a wide range of experimental conditions and comparisons [13].

This study compares the dose- dependent transcriptome data obtained for the volatile compound dimethylamine- in a pulmonary cell line (A549 cells). A549 cells were exposed to dimethylamine via an air-liquid-interface (ALI) in an in vitro system. The results from Affymetrix and TempO-Seq approaches were compared for concordance in differential mRNA expression analysis. Any differences due to technology used were identified using technique specific standard approaches. There was a high level of concordance in the dose-dependent up- and downregulation of genes measured by both techniques, as quantified by fold change (FC). However, when comparing the overlap of DEGs derived from each technique, significant differences were observed. To determine which technique more comprehensively represents the cellular response at the gene level, a subset of 269 genes was validated using RTqPCR.

## Methods

### Chemical

Chemicals were purchased at the highest purity available. Dimethylamine (DMA, purity > 99%, Dimethylamin 2.0) was purchased from Linde Gas.

### Cell culture and exposure

The A549 cell line was purchased from a commercial supplier (ATCC; LGC Promochem). Cells were routinely taken from a stock pool and grown in 75cm^2^ flasks by use of Dulbecco´s MEM medium (Seromed, Berlin) supplemented with 10% FCS and antibiotics. Cells were passaged every 3 to 4 days. During each passage beside continuous microscopic observation, cell quality and quantity were checked by use of an electronic cell counter (CASY^®^ Cell Counter + Analyser System; Schärfe System, Reutlingen, Germany). [14]

### Set-up of the air-liquid-interface cell culture system

To mimic the exposure situation of the epithelium in the in vivo lung the most common approach used is the air-liquid-interface (ALI) technique based on cell cultures on microporous membranes. Therefore, cells were initially cultivated under their cell type specific conditions in 75 cm² culture flasks using submerged conditions. Culture medium was changed every to 2–3 days. Before reaching 80% confluence, cells were subcultivated. During a cell passage an aliquot of the cells was then seeded on microporous membranes (Inserts, BD Falcon; 0.4 μm pore size; growth area ~ 1 cm²). Cells were further cultivated on the membranes for approximately 72 h until they reached a confluent monolayer as inspected by light microscopy. Serum was removed from the culture during a medium change 18 h before exposure. Previous to the exposure with the model substances, residual liquid from the apical side of each cell monolayer was gently removed. During the treatment, cells were nutrified by culture media from beneath the membrane solely while being exposed to the test substances from the top. [14]

### Cell exposure

A549 cells were exposed to dimethylamine under ALI-conditions in 12-well plates (P.R.I.T.^®^ ExpoCube^®^) [15]. Exposure took place for 60 min by applying exposure flows of 3 ml/min per 1 cm² ALI culture. The experimental design was based on 3 groups including 4 cultures / plate being exposed to the test substance, 4 cultures / plate being exposed to clean air as exposure control and the remaining wells / plate as non-exposure controls (no application of exposure flow). Vapor concentrations were set up by controlled in-line evaporation of the liquids using an impinger and dilution with clean air. Concentrations were monitored online using quantitative FT-IR analysis (Gasmet DX4000). High dose (HD) = 81.7 ppm, mid dose (MD) = 41.1 ppm, low dose (LD) = 14.9 ppm.

Exposure for one hour was followed by a 23 h recovery period before the next exposure started. Within a 72 h interval a total of 3 exposure and recovery periods were accomplished without medium change in between. For each exposure condition 3 biological replicates where tested, each was based on 2 pooled wells.

### RNA isolation

For the TempO-Seq platform RNA isolation is not mandatory but may benefit the analysis as it ensures a good quantity of free RNA in the samples. As RNA isolation is needed for Affymetrix analysis this step has been performed and purified RNA was aliquoted for further analysis. RNA was isolated and purified using the RNeasy MiniKit (Qiagen) and treated with DNase (Qiagen). RNA concentration (A260) and purity (A260/A280 ratio) were measured by spectrophotometry (NanoDrop™ 2000 Spectrophotometer, software version 1.6.198, ThermoFisher Scientific). RNA integrity number (RIN) was evaluated using an Agilent 2100 Bioanalyzer^®^ (Agilent Technologies). All RNA samples showed very good quality as indicated by high RIN values between 9.0 and 10.0. The identical RNA from each sample was examined on all three techniques.

### Transcriptome microarrays analysis

Genome-wide transcriptome analysis was undertaken using the Affymetrix GeneChip™ Whole Transcript (WT) PLUS Reagent Kit and the GeneChip™ Human Clariom™ D Arrays according to the manufacturer’s recommendation (ThermoFisher). Total RNA (100 ng) was used as a starting material for target preparation. Microarrays were subsequently washed, stained, and scanned using the Affymetrix GeneChip™ Command Console Software with .cel files as data output.

### TempO-Seq analysis

The TempO-Seq sequencing [3] has been carried out by BioClavis using the probe panel whole human transcriptome v1 which comprises 21.110 probes designed to cover all human coding genes. Quality control of the samples confirmed that at least 50ng/µl RNA were found in each sample and the initial Read Count Analysis was conducted by BioClavis (Supplement 1).

Preprocessing of raw reads including alignment was performed by BioClavis using the TempO-SeqR workflow, providing the results in count matrix format. Samples counts were then normalized using counts per million (CPM) normalization and transformed using log2 and an offset of 1. A principal component analysis (PCA) was carried out with the prcomp-function of the build-in R-Package “stats” version 4.1.1.

### Differential expression analysis

The individual dose groups and untreated controls were compared to the clean air controls. The significant differentially expressed genes (DEGs) are determined on a per condition bases (HD, MD, LD, UT) by application of the respective platform dependent techniques, as briefly described in the following.

Array data were analyzed with the Transcriptome Analysis Console (TAC) Software 4.0 (ThermoFisher). The microarray data were normalized by the robust microarray averaging (RMA) method, and subjected to quality control metrics, for instance, check of hybridization controls and visual inspection of PCA and intensity distributions. Visualization methods of gene level differential expression were employed as recommended in the TAC software. The criteria for a given gene to be a DEG on the microarray platform was p-value < 0.05. Since linear fold changes span positive and negative space, the threshold for linear fold change absolute values was set to > 2 to cover both sides.

The differential expression analysis of the normalized count data from TempO-Seq was carried out with a customized workflow in the R-statistical programming language (Version 3.6), comprising the DESeq2-package (Version 1.26.0). Read count distributions per sample as well as binomial distribution of counts per transcript were checked in preparation of contrasting. The DESeq2 normalization step was done by setting normalization factors corresponding to the CPM factors per sample. The criteria for DEGs where false discovery rate (FDR) < 0.05 and an absolute of log2 fold change (log2FC) > 0.5.

### DEG Comparison

To enable comparisons between different technologies, Probe-IDs of both the TempO-Seq and Affymetrix panels were mapped to Ensemble transcript IDs (human genome version 38). For Affymetrix the Ensembl mapping provided was used in version (na36.hg38). For TempO-Seq the probeset was aligned to the version GRCh38v100 of the Ensembl genome to find the targeted transcripts. Transcript id could then be joined to find the probe-to-probe mapping. The linear fold changes for Affymetrix were transformed to log2FC. Spearman Correlation analyses of the different platforms were performed with the stat_cor function of the ggpubr R-Package (version 0.4.0).

### Quantitative RTqPCR

Single gene expression analyses were performed using customized RT2 profiler PCR arrays (Qiagen) in a 384-well format as described in detail by Schwotzer et al. [4]. In total, 294 genes were analyzed, consisting of target genes and stable expressed reference genes from the Affymetrix and TempoSeq transcriptome data sets. cDNA synthesis was performed using the RT2 first strand kit (Qiagen). RT2 profiler PCR arrays were conducted with an RNA equivalent of 2ng using the PCR system Applied Biosystems^®^ ViiA™7 (ThermoFisher Scientific). During the qPCR an individual cycle threshold (CT) value was generated per well. Genes were categorized as “non detected” if no fragment was detected. NormFinder for R version 5; [16] was used to identify two genes with most stable expression amongst all samples to be used as normalization genes out of the included reference genes. Data analysis of exported and normalized CT values was performed based on the comparative ∆∆CT method described by Schmittgen and Livak [17]. For additional statistics a linear model described by [18] was employed using the pcr package (version 1.2.2) implementation [19].

## Results

### Initial analysis of Affymetrix and TempO-Seq data

#### Quality control

**Fig. 1:**
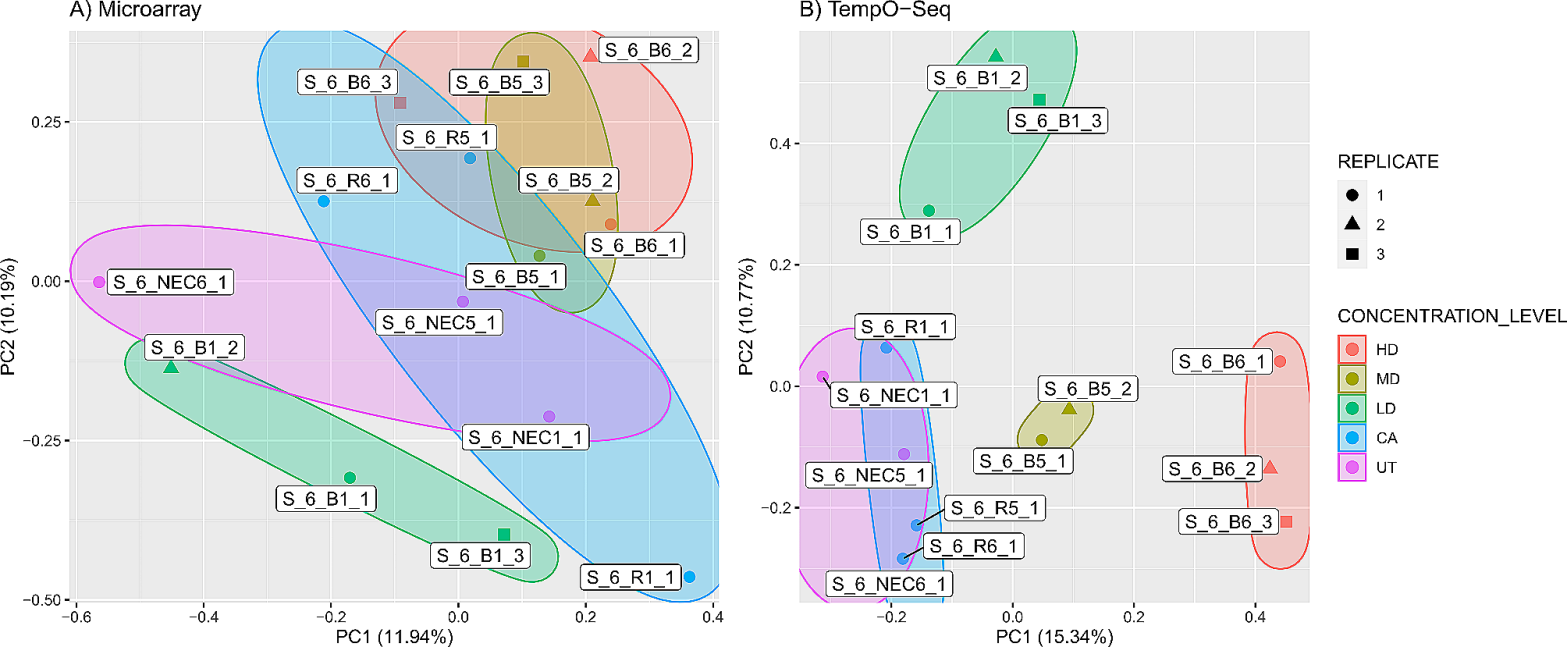
Principal component analysis (PCA) shows a separation of samples that correlates with their respective concentration levels: high dose (HD), mid dose (MD), low dose (LD), clean air control (CA), and untreated (UT). For the labelling of the individual samples a table is included in Supplement 2. One sample (S_6_B5_3) from the TempO-Seq mid dose group was removed as an outlier, due to its large distance from other samples. The outlier is not included in the plot for better visibility of the remaining samples in the PCA. See the Supplement 3 for PCA with outlier sample included. The clusters formed by the treatment conditions, (colored circles) show more overlap for the microarray experiment (**A**) compared to TempO-Seq (**B**). This indicates that the treatment-related differences among the samples are less pronounced in the microarray experiment

We used a Principal Component Analysis (PCA) on both the Affymetrix and TempO-Seq data to identify possible outliers, as shown in Fig. 1. The PCA results indicate that the variability observed in both platforms correlates with the dosed concentrations of dimethylamine (Fig. 1).

In the TempO-Seq dataset, one sample was identified as outlier in the PCA and was subsequently removed from all further analysis (Supplement 3, SF 1).

A higher overlap of samples from different dosed groups is observed in the PCA on Affymetrix data. This serves as a first indication that the treatment effect is less pronounced for microarray data (Fig. 1A) compared to TempO-Seq data (Fig. 1B). One explanation for the observed difference is the larger amount of non-coding sequences, as well as small- and precursor-microRNAs on the Affymetrix chip (see below Table 1). This assumes that coding genes are more affected by the treatment compared to other sequences.

However, the trend is the same for both platforms. The PCA shows a clear clustering of samples by tested concentration, resulting in a separation of samples in a concentration- dependent manner as the dose increases (Fig. 1A and B). Untreated and clean air controls form a joint group, indicating that there are minimal changes at the gene level due to treatment with clean air.

#### DEG Analysis

The analysis of Affymetrix microarrays showed significant DEGs for UT, LD, MD, and HD relative to air control. The fold change for individual DEGs - generally increased or decreased in a dose-dependent manner. In the HD group, 1421 DEGs (694 up / 727 down) had a p-value below 0.05 and an absolute linear fold change > 2. This represents about 1% of the measured probes. The MD group had 1029 DEGs (414 up / 615 down), while the LD group had 1196 DEGs (605 up / 591 down). The UT showed 977 DEGs (570 up/ 407 down), but these were later discarded because they were not found in the overlap of probes between the two platforms (Table 1 below). The counts of DEGs within the overlap are shown in Table 2 below. Using the FDR-criteria for DEGs analogous to the TempO-Seq analysis, only 59 sequences in the HD condition were found. As a result, no multiple test correction was applied in the Affymetrix selection of DEGs, which is consistent with the default criteria in this version of the TAC software. The analysis of TempO-Seq data revealed an average of 6.2 million counts per sample. When comparing samples treated with dimethylamine at different concentration levels to air control samples, there was an increase in the total number of DEGs with increasing test concentration.

In the HD group, 587 DEGs (325 up / 262 down) were identified, representing 2% of measured sequences. In the MD group, 65 (30 up / 35 down) DEGs were found -, while in the LD group, there were 61 DEGs (23 up / 38 down). In the UT group, 4 downregulated DEGs where found. DEGs from the UT group were not to be considered DEGs in any other dose group, and the same 4 genes were also not found to be DEGs in any of the other groups. The number of DEGs in each condition was almost equally split between up- and downregulated genes.

### Concordance of DEGs within the Affymetrix and TempO-Seq data

The number of probes and the sequence segments measured differed significantly between the two sequencing techniques. Thus, probes may have different specificities when matching a given transcript and number of variants. The probe panels of the Affymetrix and TempO-Seq technologies showed a 1-to-N mapping of the probe-sequence to Ensembl transcript ID, meaning one probe can capture multiple transcripts, typically from the same gene. Both panels were joined using Ensembl IDs and consolidated by keeping only unique probe-to-probe mappings. A total of 20,533 probes were identified that target the same 18,037 transcripts in both systems. As shown in Table 1, these 20,533 mapped probes represent the full set of comparable measurements.

The Affymetrix probeset includes additionally 52,902 Non-coding genes, 2129 precursor-microRNA, 1975 small RNA, 404 ribosomal RNA and 6 tRNA, which are not referenced by Ensembl transcript IDs (Table 1). Consequently, they cannot be mapped to the TempO-Seq panel.

**Table 1:** Table of probe labels and overlap of both platforms Affymetrix and TempO-Seq. The largest overlap of probes is found with the label of Multiple_Complex and coding genes

probelabel	No. of probes Affymetrix	No. of probes TempO-Seq	No. of probes mapped to Ensembl ID (Affymetrix)	No. of ensembl transcripts covered by Affymetrix	No. of probes mapped to Ensembl (TempO-Seq)	No. of ensembl transcripts covered by TempO-Seq panel	Overlapping probes (*N*-to-*N*)
Coding	18,858		3846	7422	3339	5230	3589
Multiple_Complex	29,510		24,729	145,197	16,445	38,763	16,824
Non-coding	66,845		13,943	19,621	19	33	31
Precursor_microRNA	3297		1168	1168	-	-	-
Pseudogene	4340		3573	3613	62	89	89
Ribosomal	507		103	103	-	-	-
Small_RNA	2386		411	414	-	-	-
tRNA	6		-	-	-	-	-
Unassigned	10,001	21,110	486	491	-	-	-
Total	135,750	21,110	48,259	178,029	19,865	44,115	20,533

The two platforms were compared based on shared measured probes corresponding to the above described 20,533 mapped probe IDs. In this dataset, Affymetrix reported 414 DEGs with 267 upregulated and 147 downregulated. TempO-Seq showed 584 DEGs with 330 upregulated and 254 downregulated. Only 153 DEGs intersected between the two platforms with 95 upregulated and 58 downregulated. This corresponds to 37% and 26% of the DEGs measured for Affymetrix and TempO-Seq, respectively (Table 2).

**Table 2:** Overlap and union of DEGs in HD condition for both Affymetrix and TempO-Seq platforms

		Affymetrix	TempO-Seq	Overlap Affymetrix – TempO-Seq	All DEGs
Whole genome (20,533 genes)	total	414	584	153	845
	up	267	330	95	454
	down	147	254	58	295
	conflicting				96

**Fig. 2:**
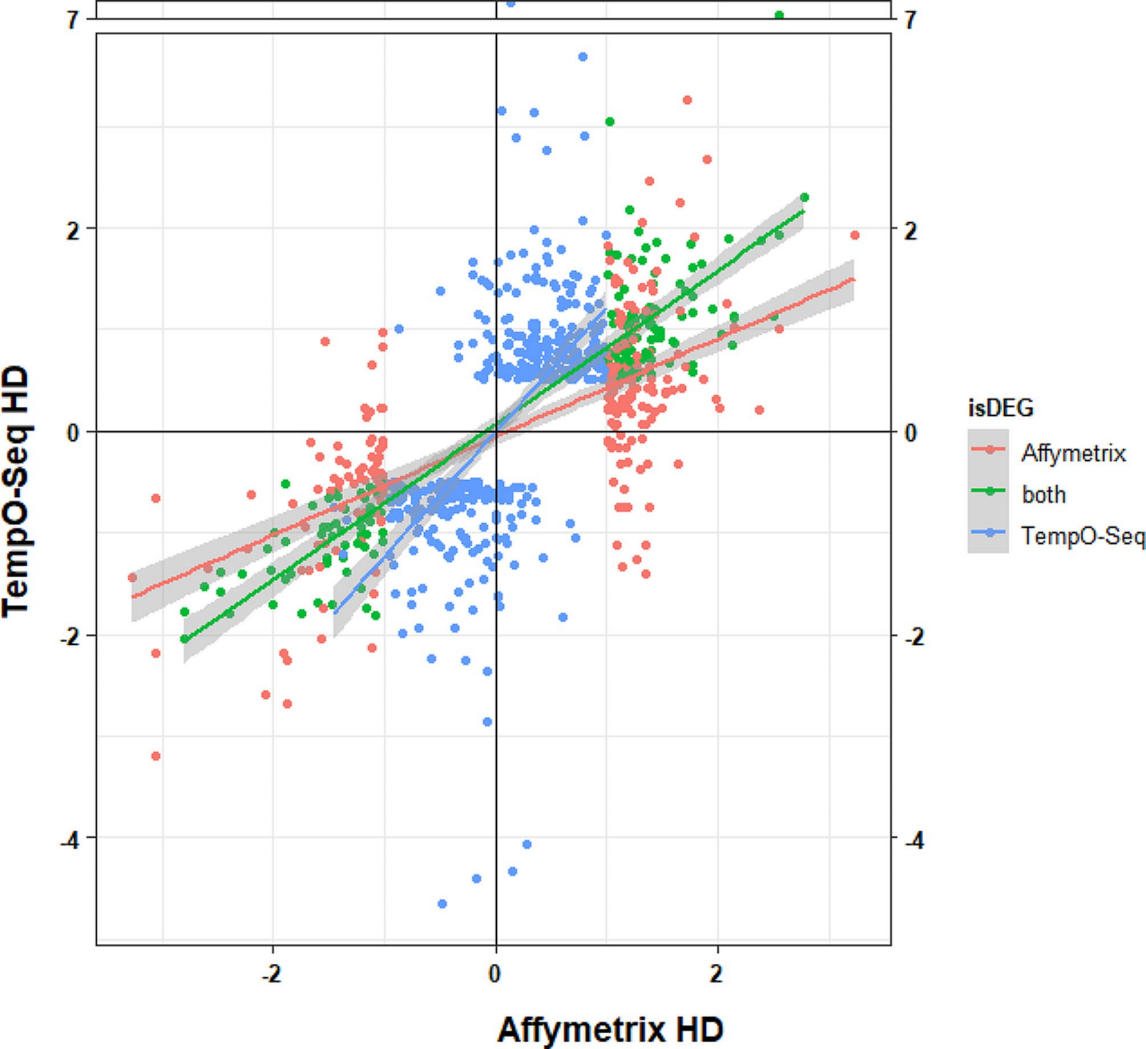
Scatterplot of the log2 transformed fold changes for genes differentially expressed in Affymetrix and/or TempO-Seq at the HD: red – DEG in Affymetrix only; blue – DEG in TempO-Seq only, green – DEG in both platforms. DEGs significant in both platforms (green) show consistency in terms of direction. Whereas some DEGs of either platform (red & blue) can be found expressed in opposing direction (top-left, bottom-right) albeit not being significant for one platform or direction

A correlation of fold changes from all obtained DEGs, stratified by their origin (either Affymetrix, TempO-Seq, or consistent across both) show high concordance in terms of up- and downregulation within both platforms. The majority of DEGs (88.6%) fall in the bottom left (downregulated) and top right quadrant (upregulated) of the plot, with few conflicting DEGs (11.36%, *N* = 96; top right and bottom left quadrant, Fig. 2; Table 2).

### Validation of the platforms DEGs using RTqPCR

The small number of overlapping DEGs in both platforms motivated us to investigate the “true number of DEGs” more closely. For this analysis, we used RTqPCR to validate DEGs in a smaller dataset of 294 genes. These genes represent (i) DEGs in both platforms at HD (*N* = 91, geneset I), (ii) DEGs only in the Affymetrix platform (*N* = 33, geneset II) or in the TempO-Seq platform (*N* = 122, geneset III) and (iii) genes which are not DEGs in either platform (*N* = 48), including reference genes for normalization. Four genes of geneset I and II, as well as four from geneset III, were not detected in RTqPCR. The dataset of DEGs in either TempO-Seq or Affymetrix with validation data totals 238 DEGs, excluding not detected (n.d.) genes.

### Dose dependency

**Fig. 3:**
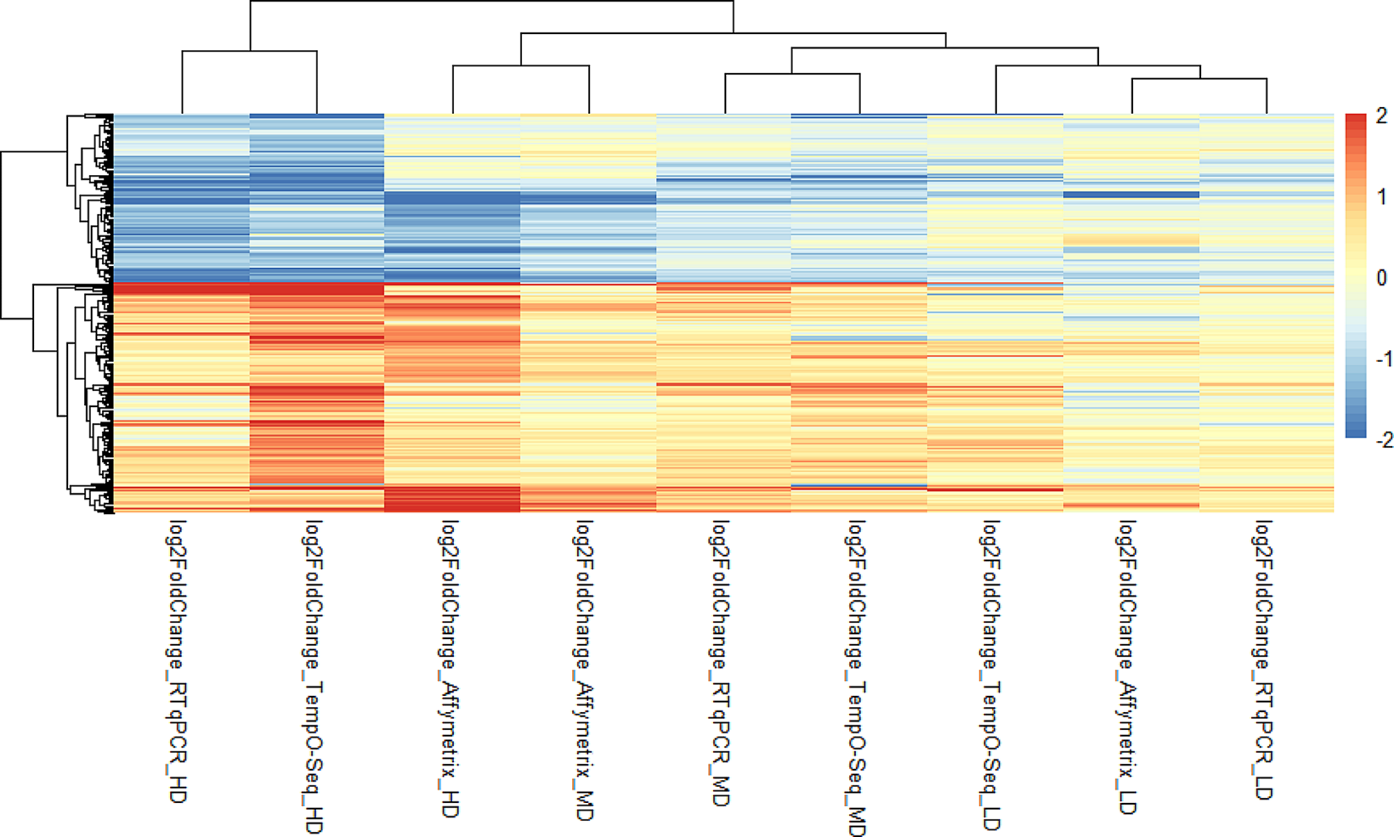
The heatmap shows the log2 fold changes for 238 genes, which have been shown to be differentially expressed in either the Affymetrix or TempO-Seq platforms. Generally, both platforms show similar expression direction with the lower dose showing little change overall with 28 genes being significantly differentially expressed

The effect of dose on the validation geneset was assessed using three sub- cytotoxic dose levels. The genes that were found to be differentially expressed at the low dose (LD) were largely also found to be DEGs in the MD and HD conditions. The largest absolute fold change value for these genes, indicating the most pronounced effect, was found at HD condition. Figure 3 displays a dose-dependent increase in DEGs and their associated log2FC values per platform.

The stronger color intensity in the heatmap indicates that the HD conditions can be distinguished due to their high fold change values. The dendrogram at the top of the heatmap shows the distance between the different conditions, as determined by hierarchical clustering with complete linkage. While the LD and MD conditions cluster well across the different techniques, the Affymetrix HD shows a slight deviation from its group. Since the HD effects are most pronounced and consistent with what is observed in MD and LD conditions, we focused on validating the HD condition.

### Confirmation of DEGs by RTqPCR

**Fig. 4:**
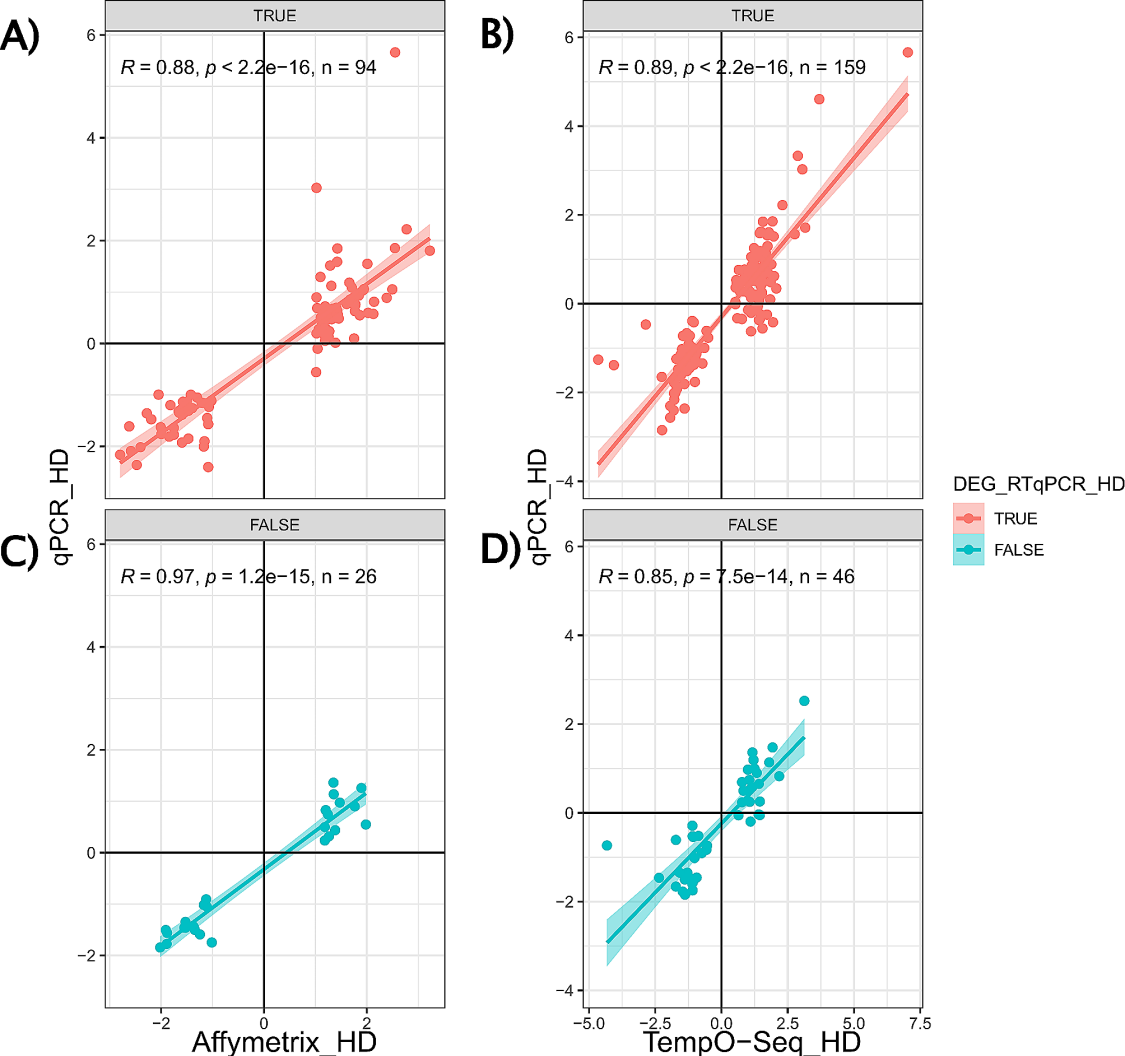
The scatterplots show DEGs of the two platforms for Affymetrix (*n* = 120) and TempO-Seq (*n* = 205) stratified for being DEG at HD in RTqPCR platform. The individual panels show (**A**) Affymetrix DEGs with significance in RTqPCR, (**B**) TempO-Seq DEGs with significance in RTqPCR, (**C**) Affymetrix DEGs without significance in RTqPCR, and (**D**) TempO-Seq DEGs without significance in RTqPCR validation. Correlation scores are shown as R in the scatterplots. Correlations between the fold changes of the individual platform with the validation dataset of 238 genes comparing the two test platforms

The dynamic range, as expressed by log2FC values, varies between the Affymetrix, TempO-Seq, and RTqPCR techniques. The scale limits for TempO-Seq range from − 5 to 7.5, while those for RTqPCR from − 2 to 6. In contrast, the values for Affymetrix range from between − 3 and 3, suggesting a lower dynamic range for the Affymetrix platform.

This effect is exemplified by the gene *IL1RL1*. In RTqPCR and TempO-Seq, *IL1RL1* shows the highest upregulation with 5.6 log2FC and 7 log2FC, respectively. In Affymetrix, it is among the most upregulated with a 2.5 log2FC. Despite these quantitative differences in fold change, *IL1RL1* it is detected as DEG in all three techniques.

The validation dataset comprises 238 genes, excluding those n.d., which are DEGs in one of the two sequencing platforms. Of these 238 DEGs, 186 were initially confirmed as DEGs by RTqPCR in the HD group before identifying 27 genes with conflicting expression direction. A total of 159 DEGs were confirmed to have a similar fold change direction across all three systems (Table 3). However, 11 DEGs had an opposing fold change direction determined by RTqPCR in all systems. Additionally, for 9 genes, the fold change direction determined by RTqPCR did not show the same direction change as Affymetrix, and for 7 genes, it did not show the same direction change as TempO-Seq.

Of the 238 DEGs in the validation set, Affymetrix contributed 120 genes from genesets I and II. RTqPCR confirmed 92 of these DEGs, while 2 DEGs showed the opposite fold change (conflicting), and 26 were not differentially expressed (not confirmed).

TempO-Seq contributed 205 genes from genesets I and III to the validation set of 238 DEGs. RTqPCR confirmed 144 DEGs, while 15 DEGs showed the opposite fold change, and 46 were not differentially expressed (not confirmed).

Of the 87 genes in geneset I (both platforms), 66 were confirmed by RTqPCR. One gene showed a similar direction between Affymetrix and TempO-Seq, but the opposite direction for RTqPCR at HD condition. A total of 20 genes were not confirmed as DEGs in RTqPCR at HD condition. However, 16 of these were DEGs in either the MD or LD conditions for the RTqPCR validation.

The fold changes for both platforms shows very good correlation coefficients of 0.88 to 0.89 with the RTqPCR validation (Fig. 4A, B). A good correlation of the fold changes is also observed for genes that were differentially expressed in either Affymetrix or TempO-Seq but were not confirmed by RTqPCR as significantly changed (Fig. 4, C and D).

Despite the high correlation coefficients, some genes considered to be differentially expressed in either platform show fold changes of opposite direction in the RTqPCR validation. This is seen for 2 genes that were upregulated in Affymetrix but downregulated in RTqPCR (Fig. 4A), and for 15 genes in TempO-Seq with the same trends (Fig. 4B).

Pathway enrichment analyses are often performed using a shortlist of “top” regulated DEGs. However, a comparison of three different datasets demonstrates that the absolute value of FC alone is not always a reliable descriptor for ranking DEGs. For example, the gene *IL1RL1* has a very low absolute expression in control samples, with 4 log intensity (Affymetrix), 9.5 mean CPM (TempO-Seq), and 35 ct (RT-qPCR). As a result, - a slight increase in expression levels due to treatment, can easily result in a higher fold change. On the other hand, genes with already high expression levels in controls, such as *S100A6*, which has a log intensity value of 10 in Affymetrix, 80,000 mean CPM in TempO-Seq, and 16 ct in RTqPCR, require a relatively large change in expression to achieve a significantly high (in our study 1.5) or similar fold change. Therefore, in addition to p-values and fold change, base expression should also be considered when selecting genes of interest, taking into account platform differences.

**Table 3:** Differentially expressed genes in HD condition of Affymetrix and TempO-Seq platforms confirmed by RTqPCR validation: the columns show the validation gene set, DEGs from the two platforms Affymetrix and TempoO-Seq and the overlap of both

	Union of DEGs (*N* = 246)	DEG Affymetrix (*N* = 124)	DEG TempO-Seq (*N* = 213)	DEG Affymetrix + TempO-Seq (*N* = 91)
Confirmed DEG*	159 (64.6%)	92 (74.2%)	144 (67.6%)	66 (72.5%)
DEG with conflicting FC**	27^#^	2	15	1
Not confirmed***	52	26 (22%)	46 (22%)	20
Not detected	8	4	8	4

*Confirmed – DEG with significant difference to control determined by p-value is given for the corresponding platforms and RTqPCR; the direction of the differential expression, expressed as FC (up or down regulated) is similar.

**conflicting FC - DEGs with opposite FC directions.

***Not confirmed: Genes which do not show significant differential expression in RTqPCR are labelled as not confirmed.

^#^Subset includes DEG with similar FC direction in Affymetrix and TempO-Seq but opposite FC observed in RTqPCR (*N* = 11); union DEGs with similar FC direction in RTqPCR and TempO-Seq but conflict for Affymetrix (*N* = 9) and union DEGs with similar FC direction in RTqPCR and Affymetrix but conflict in TempO-Seq (*N* = 7).

These results indicate that both the Affymetrix and TempO-Seq platforms detected DEGs with some margin of error. To summarize, 24% of the 246 genes were either not confirmed or not detected by RTqPCR. Additionally, 11% of the genes showed conflicts in the direction of fold change. Of these, RTqPCR conflicted with the fold changes of DEGs that agreed between Affymetrix and TempO-Seq (*n* = 11; 1 from geneset I, 1 from geneset II, 9 from geneset III). Furthermore, RTqPCR could not confirm the direction of fold change for Affymetrix (*n* = 9; 9 from geneset III) and for TempO-Seq (*n* = 7; 2 from geneset II, 5 from geneset III).

A minority of 26 DEGs for the Affymetrix platform and 46 DEGs for the TempO-Seq platform were not confirmed by RTqPCR. Of these, 2 and 15 genes had significant changes but in opposing direction to RTqPCR. The overlap of Affymetrix and TempO-Seq shows that one gene (HMGA1) was agreed by both platforms but showed opposite fold change direction in RTqPCR. A total of 20 genes were concordant in Affymetrix and TempO-Seq but could not be confirmed by RTqPCR.

An inspection of the individual gene expression values showed similar fold changes for genes overlapping in the two platforms which were not confirmed by RTqPCR (*n* = 20; Table 3). These genes showed similar response to dose in terms of fold change and often a significant change for MD in RTqPCR, but the significance was not achieved at HD in the RTqPCR platform. These genes are shown in the supplement (Supplement 3, SF 2). To better understand the inconsistencies a closer look at individual genes is shown in the next section, highlighting the different types of conflicts between the three techniques by example of individual gene expression values.

**Fig. 5:**
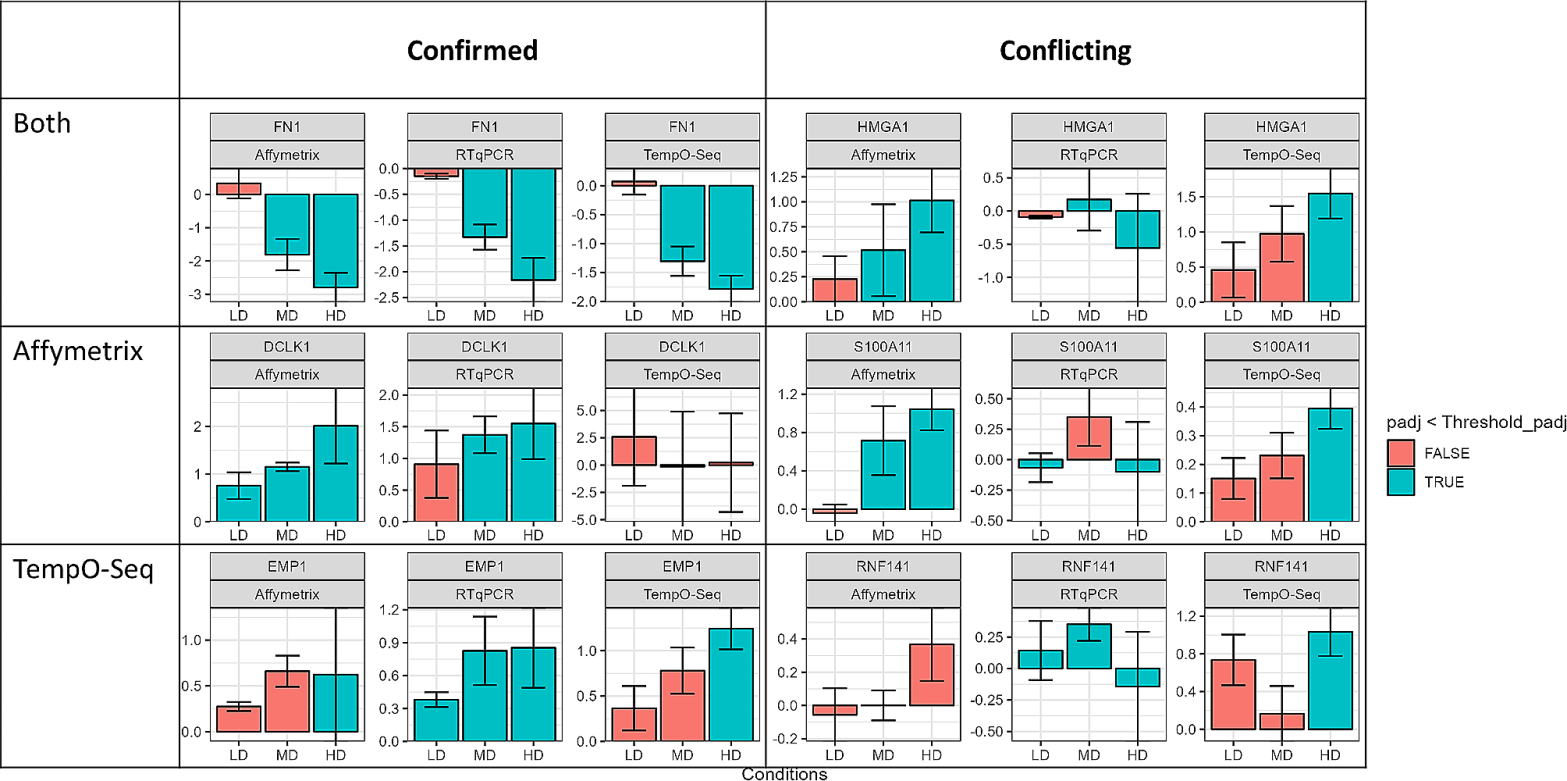
Comparison of individual gene expression for a selection of genes between all systems. The bars represent the log2 fold change for selected genes at LD, MD and HD. This selection exemplifies confirmation of DEGs using RTqPCR as well as conflicting results. Padj refers to p-value for Affymetrix and RTqPCR and to FDR for TempO-Seq (< 0.05)

Genes can exhibit differences in fold change and significance across the different platforms. This observation is exemplified in Fig. 5, which compares the individual expression values for a selection of genes in Affymetrix, TempO-Seq and RTqPCR. The column for genes confirmed by RTqPCR displays expression values for genes that were concordant between platforms and confirmed, as well as genes individually detected and confirmed by each platform. While not all differences in gene expression are shown, three distinct cases are highlighted: poor probe design, values just below an arbitrary cutoff, and high data variability.

For *FN1*, all three systems agree on a dose-dependent downregulation, which is shown to be significant starting with the MD. For *DCLK1*, a gene detected by Affymetrix and confirmed with RTqPCR, TempO-Seq showed no significant differential expression. The low number of counts for this gene in the TempO-Seq data suggests that the design of the probe by BioClavis is flawed. *EMP1* shows high concordance in all three systems, with all showing a significant dose-dependent upregulation, except for the HD in Affymetrix where high variability was observed. Similar variability can be observed for conflicting genes in the second column of Fig. 5. Similar trends can be observed for LD and MD with RTqPCR showing unexpected downregulation at HD with large error bars. In addition to not being confirmed at HD by RTqPCR, *S100A11* shows an upregulation of 0.4 log2FC, which is just below the cutoff for DEGs in TempO-Seq (log2FC > 0.5). *S100A11* showed an average signal (log2) of 14.7 in Affymetrix and a mean normalized count of 22,117 in TempO-seq for the CA samples, considering the high absolute expression of *S100A11*, this gene needs to be evaluated for its biological relevance, despite the low observed fold change.

Another reason for the low direct overlap of DEGs in both platforms might be due to different isoforms of genes being targeted. In the analysis probe signals are summarized to a gene level. In this summarization different sets of gene isoforms may be considered. This is exemplarily illustrated for the gene DCLK1.

The gene DCLK1 has a total of 8 transcript annotations in ensembl GRCh38.

**Fig. 6:**
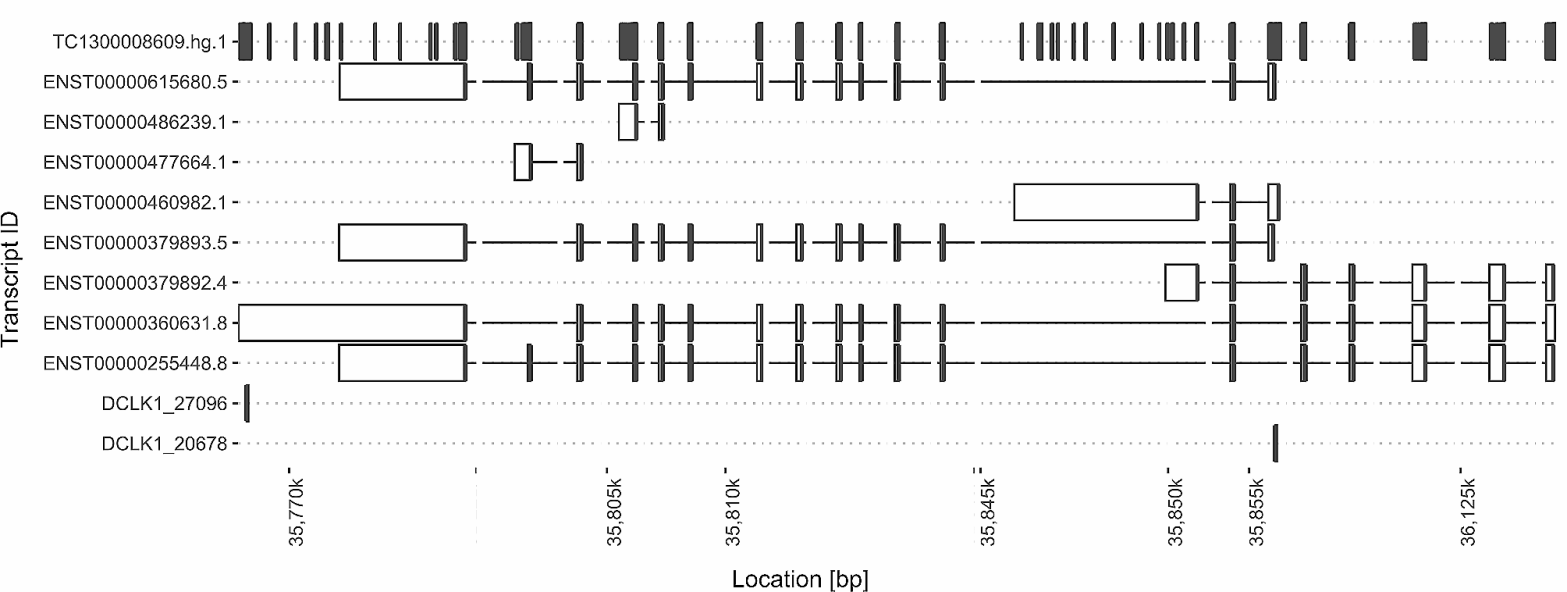
Individual transcripts of DCLK1 and alignment sites of TempO-Seq probes DCLK1_27096 and DCLK1_20678. The Affymetrix probes for DCLK1 were combined on one row at under TC1300008609.hg.1. Some Affymetrix probes align to multiple sites within the DCLK1 gene, the first occurrence per probe is indicated in the plot. The current version (.5) of ENST00000615680 is no longer covered by the TempO-Seq probe

The TempO-Seq probe-set matches 3 transcripts for DCLK1. Two probe signals (DCLK1_20678 and DCLK1_27096) are combined for the read-out of DCLK1. Both probes perfectly match to exon-regions of the ENST00000615680.4 transcript (not shown). Each probe additionally matches one ensembl-transcript of this gene namley ENST00000460982.1 (Exon ENSE00001931884) and ENST00000360631.8 (Exon ENSE00003737572) respectively. Five transcripts out of 8 total annotated for DCLK1 are not covered by the TempO-Seq probes (Fig. 6).

The Affymetrix array contains 265 probes that target DCLK1 and possible exon-exon junctions. In the Affymetrix analysis the signal of these probes is summarized to one gene level, however intensities indicate that individual exons of 7 transcripts are not detected in any condition, leaving ENST00000477664.1 as candidate to drive the high up-regulation of DCLK1.

Both platforms target different regions of a gene. Depending on splicing, a specific gene isoform can be differently abundant. In the presented data the upregulation of DCLK1, observed in Affymetrix is clearly driven by a gene variant, not covered by TempO-Seq. A better overlap between the two platforms could be archived by discarding Affymetrix probes with the consequence of both platforms not accurately representing DCLK1 expression.

## Discussion

Transcriptome data are widely generated and used in toxicological research to assess pathways and mechanisms that lead to adverse outcomes. This resulted in the rapid increase in sample and data volumes, through research initiatives such as ToxCast [20], RiskHunt3r [21], PrecisionTox [22] and PARC [23]. Furthermore, the significant reduction in sequencing costs in recent years, along with the development of new high throughput technologies such as TempO-Seq, has contributed to an increase in data. As a result, there is great interest in comparing and integrating datasets into subsequent analyses.

Integrating “omics” data from different platforms requires a good understanding of their similarities, differences, and their associated uncertainties. In this study, we compared the TempO-Seq and Affymetrix platforms and validated a subset of genes using RTqPCR assays on identical RNA samples. The aim of this comparison was not to exclude either platform for use in toxicology, but to carefully examine their differences.

The Principal Component Analysis (PCA) showed that the treatment influenced individual samples. Samples with similar treatment conditions clustered together and were distinguishable along the first principal component. However, the separation of samples by treatment condition was less pronounced for the Affymetrix platform. This could be due to many more probes that contribute to the variability of the Affymetrix Clariom D samples. Since most of these probes are not affected by the treatment, they add noise to the PCA. Additionally, even affected genes may contribute little to the separation due to the lower dynamic range achieved by fluorescent-based read-out of the microarray technique.

In our analysis, a high dynamic range was achieved using TempO-Seq. This is a commonly observed benefit of RNA-Seq compared to microarray-based techniques. The higher dynamic range allows, for example, for more accurate evaluation of dose responses.

One major difference between the tested platforms is the large number of non-coding transcripts captured within the Affymetrix Clariom D panel target. Formerly, the ability to detect novel sequences was an advantage of using RNA-Seq over microarray-based transcriptomics analysis, such as Affymetrix. In the meantime, the human Clariom D panel represents all non-coding and coding sequences identified by RNA-Seq. The TempO-Seq platform employs a targeted approach through probe hybridization step to reduce cost per sample, simplify library preparation, and enable high throughput. Despite using an RNA sequencing at its core, the probe set for coding genes in a whole-genome analysis generated by TempO-Seq is more limited compared to the Affymetrix Clariom D panel. In addition to the scope of probe coverage of genes, differences in the length and specific target sequence of probes exist. While TempO-Seq aims to target multiple isoforms of a gene with a single probe, Affymetrix has extended its probe-set in newer panels to include non-coding regions, junctions and predicted transcript regions. The inclusion of control and mismatch probes results in an increase of complexity in the analysis. Still the Affymetrix platform identified fewer DEGs compared to TempO-Seq (414 / 584), due to the lower dynamic range of florescence-based techniques and noise resulting from shorter and less specific probes. Affymetrix is not able to pick up signals of low abundant genes.

At the moment analysis of alternate splicing events is not possible using the TempO-Seq technology as no probes are included to target specific junction regions.

As exemplarily shown for the DCLK1 some probes require optimization to cover all transcript variants and BioClavis is continuously improving and updating the TempO-Seq probe panel. While the ensembl reference genome - transcript annotations are being updated as well. With the update of ENST00000615680.4 to ENST00000615680.5 in the latest ensembl version, the two DCLK1 probes no longer target this transcript sequence.

The number of measured probes can influence the analysis outcome because statistics such as adjusted p-values are affected by the number of probes when multiple testing is considered [24]. After identifying DEGs, we applied a correction for multiple testing to the TempO-Seq dataset to determine significant changes. But this correction is not part of the standard analysis of the much larger Affymetrix Clariom D probe panel. Since this study only considers the coding genes of the Affymetrix Clariom D probe panel, it could be useful to recalculate adjusted p-values for only mapped probes. We did not perform this adjustment using Benjamini-Hochberg correction because we wanted to compare DEGs derived using typical standard criteria for each procedure.

The comparison of the fold change of DEGs between the two investigated platforms Affymetrix and TempO-Seq, showed a high concordance. In the HD condition, about 89% of all DEGs agree in the direction of fold change. However, only 18% of these DEGs are considered significant by both platforms, representing 26% and 34% of DEGs for Affymetrix and TempO-Seq, respectively. A similar trend was observed across different concentration levels.

At LD condition, the directions of fold change also showed a high correlation, but for fewer DEGs as the overlap shrank to only 10 DEGs out of a total of 216 DEGs (165 DEGs for Affymetrix and 61 DEGs for TempO-Seq). Despite the high concordance in regulation of genes, the number of identified DEGs varied between the two platforms.

In order to archieve a higher overlap between the two platforms a comparison based on target regions of probes could be used, this however would require highly customized analysis workflows for each platform.

The p-values for each platform were used to determine if there was a significant difference from the control. The outcome was then used to compare the platforms. For the validation using RTqPCR, the significance of difference for a gene compared to control is not as critical as the direction and size of expression change. RTqPCR results for validation are commonly accepted without statistical analysis. The high similarity of expression in the 20 genes concordant between Affymetrix and TempO-Seq, but not significantly confirmed by RTqPCR could be explained by the different analysis strategies used for each system. These differences are determined by the underlying techniques used, such as the binomial distribution of NGS data versus the linear model employed for array-based and PCR- based techniques. In this study, the goal was not to harmonize analysis strategies, but to compare the results of differential expression analysis as it would be the preferred approach for each given platform.

Our findings are consistent with previous publications, which reported a low overlap of DEGs when the effects of treatment are minimal. For example, 12.5% overlap of DEGs was observed for the compound diclofenac across the microarray and RNA-Seq platforms, with a total of 210 DEGs. In contrast, a much larger overlap of 78.5% was observed for the more active compound carbon tetrachloride, which induced 2,275 DEGs in both platforms [12].

Our analysis of dimethylamine showed an overlap of 153 DEGs (18%) for a total of 845 DEG across the two platforms (Table 2).

The Spearman correlation between the two platforms was *R* = 66% (Fig. 2). This correlation is negatively affected by genes with non-significant changes from either platform due to the union combination. The correlation of overlapping genes showed, however, a higher correlation coefficient of 89% (Supplement 3, SF 3). This finding is also in agreement with a recent study of Rao and colleagues [12], in which the correlation coefficients increased for overlapping DEGs compared to unfiltered fold changes from *R* = 44–67% to 60–83%.

The comparison presented in this paper is based on a more evenly weighted differential expression analysis as compared to the data of Rao et al. The ratio of DEGs found in Affymetrix over the number of DEGs in TempO-Seq in our data was closer to 0.7, compared to 10 (DEGs in RNA-Seq over DEGs in microarray) for the comparison of microarray with RNA-Seq published by [12]. In the study of Rao et al. the GeneChip Rat Genome 230 2.0 Array was used. This microarray had captured only about a tenth of the DEGs captured with RNA-Seq. Whilst in the present study the number of DEGs found with the Affymetrix Clariom D probe panel and TempO-Seq whole transcriptome probe panel is much closer to a ratio of 1.

Like previous studies [12, 25], our analysis show that the concordance of two platforms depends on the transcript abundance and additionally highlight the importance of selecting a probe-set covering the biological space fit for the scientific question. It is also important to use the same version of the reference genome for mapping probe signals when comparing platforms.

The validation system using RTqPCR to confirm the overlap of DEGs showed that 22% of results from either platform were false positives. Of the genes selected for Affymetrix, 76% were confirmed, while 70% of genes were confirmed for TempO-Seq. Even among the overlap of DEGs from both Affymetrix and TempO-Seq, only 76% were confirmed, suggesting some uncertainty inherent in RTqPCR. None of the platforms could be validated to 100% by RTqPCR, and some probability of false positive is expected for any platform. The variable results per platform indicate a need for better standardized analysis frameworks for application of transcriptomics data in toxicology. As observed in the correlation of fold changes between the Affymetrix and TempO-Seq platforms, the directions for most DEGs agree and often the amounts coincide. Similar findings are presented in a technical note by Affymetrix which showed a good correlation of fold changes between Affymetrix and RTqPCR validation [26, 27].

Since the criteria for statistical significance differ between platforms and yield different DEGs, standardizing the analysis approach would be beneficial for achieving more consistent results, better reproducibility, and the ability to integrate various datasets, such as those for a chemical stressor. Additionally, transparent mapping of probes to target regions, harmonizing the probe designs, which differ between the two platforms and the RTqPCR technique, could help improve concordance.

Further pathway analysis will improve our understanding of the relevance of changes in gene transcription. Rao et al. showed that there was an improved concordance when comparing platforms on a pathway level instead of the DEGs. This is expected to some degree, as known pathways tend to feature well studied genes or gene products, which are more likely to have been reproducibly measured in the past. Therefore, they are more likely to yield concordant results in a platform comparison.

In other words, pathway analyses are inherently biased by previous knowledge towards genes with little baseline variability. Moreover, the results of pathway analysis results depend on the set of pathways chosen. Less biased techniques include weighted-gene correlation networks (WGCNA), which can be used to extract more biologically interpretable results in future analysis.

## Conclusion

By using these very sensitive techniques, differences were observed by performing analyses on different platforms. Each of the three systems used, yielded additional DEG candidates with statistical significance. Nonetheless, only about 10% of the detected genes in the overlap of Affymetrix and TempO-Seq were inconclusive due to conflicting results.

For a very accurate measure, the low-throughput technique RTqPCR is still needed for validation. The requirements for a platform will vary depending on the research question, from high-throughput covering many genes for screening to covering more specifically selected genes (in many testing conditions) for regulation.

TempO-Seq can enable targeted sequencing for dose-response analysis with high-dynamic range in a cost-effective manner for testing many compounds/samples. For identification of new pathways and processes driven by transcripts (including miRNA), platforms with appropriate probe panels or capable of discovering novel transcripts are still imperative.

In toxicology there is a requirement for a vast amount of data given the size of the chemical domain, the need to incorporate concentration ranges, as well as several cell lines in the case of in vitro testing. Thus, strategies need to be developed to integrate different types of data.

### Electronic supplementary material

Below is the link to the electronic supplementary material.

Supplementary Material 1

Supplementary Material 2

Supplementary Material 3

## Data Availability

The datasets supporting the conclusions of this article are available in the Gene Expression Omnibus (GEO) repository, under GEO accession number GSE240878 (https://www.ncbi.nlm.nih.gov/geo/query/acc.cgi?acc=GSE240878).

## References

[1] Merrick BA (2019). Next-generation sequencing data for use in risk assessment. Curr Opin Toxicol.

[2] Cherianidou A, Seidel F, Kappenberg F, Dreser N, Blum J, Waldmann T (2022). Classification of Developmental toxicants in a human iPSC Transcriptomics-based test. Chem Res Toxicol.

[3] Yeakley JM, Shepard PJ, Goyena DE, VanSteenhouse HC, McComb JD, Seligmann BE (2017). A trichostatin A expression signature identified by TempO-Seq targeted whole transcriptome profiling. PLoS ONE.

[4] Suzuki S, Furusawa C, Ono N, Kashiwagi A, Urabe I, Yomo T (2007). Insight into the sequence specificity of a probe on an affymetrix genechip by titration experiments using only one oligonucleotide. Biophysics.

[5] Toxicology in the 21st Century [Internet] Maryland (USA). National Institutes of Health; [ https://tox21.gov/.

[6] Huang R, Xia M, Sakamuru S, Zhao J, Lynch C, Zhao T (2018). Expanding biological space coverage enhances the prediction of drug adverse effects in human using in vitro activity profiles. Sci Rep.

[7] Huang R, Xia M, Sakamuru S, Zhao J, Shahane SA, Attene-Ramos M (2016). Modelling the Tox21 10 K chemical profiles for in vivo toxicity prediction and mechanism characterization. Nat Commun.

[8] Harrill J, Shah I, Setzer RW, Haggard D, Auerbach S, Judson R (2019). Considerations for strategic use of high-throughput transcriptomics chemical screening data in regulatory decisions. Curr Opin Toxicol.

[9] Bushel PR, Paules RS, Auerbach SS (2018). A comparison of the TempO-Seq S1500 + platform to RNA-Seq and microarray using Rat Liver Mode of Action samples. Front Genet.

[10] Lewis RW, Hill T, Corton JC (2020). A set of six gene expression biomarkers and their thresholds identify rat liver tumorigens in short-term assays. Toxicology.

[11] Li D, Gong B, Xu J, Ning B, Tong W (2021). Impact of sequencing depth and Library Preparation on Toxicological interpretation of RNA-Seq Data in a three-sample scenario. Chem Res Toxicol.

[12] Rao MS, Van Vleet TR, Ciurlionis R, Buck WR, Mittelstadt SW, Blomme EAG (2018). Comparison of RNA-Seq and microarray gene expression platforms for the Toxicogenomic evaluation of liver from short-term rat toxicity studies. Front Genet.

[13] Costa C, Giménez-Capitán A, Karachaliou N, Rosell R (2013). Comprehensive molecular screening: from the RT-PCR to the RNA-seq. Translational Lung Cancer Res.

[14] Ritter D, Knebel J (2014). Investigations of the Biological effects of Airborne and Inhalable substances by Cell-based < i > in Vitro methods: fundamental improvements to the ALI Concept. Adv Toxicol.

[15] Ritter D, Bitsch A, Elend M, Schuchardt S, Hansen T, Brodbeck C (2018). Development and evaluation of an in Vitro Test System for Toxicity Screening of aerosols released from Consumer products and First Application to aerosols from a hair straightening process. Appl Vitro Toxicol.

[16] Andersen CL, Jensen JL, Ørntoft TF (2004). Normalization of real-time quantitative reverse Transcription-PCR data: a model-based Variance Estimation Approach to identify genes suited for normalization, Applied to bladder and Colon Cancer Data sets. Cancer Res.

[17] Schmittgen TD, Livak KJ (2008). Analyzing real-time PCR data by the comparative C(T) method. Nat Protoc.

[18] Yuan JS, Reed A, Chen F, Stewart CN (2006). Statistical analysis of real-time PCR data. BMC Bioinformatics.

[19] Ahmed M, Kim DR (2018). Pcr: an R package for quality assessment, analysis and testing of qPCR data. PeerJ.

[20] Dix DJ, Houck KA, Martin MT, Richard AM, Setzer RW, Kavlock RJ (2007). The ToxCast program for prioritizing toxicity testing of environmental chemicals. Toxicol Sci.

[21] RISK-HUNT3R -. RISK assessment of chemicals integrating HUman centric Next generation Testing strategies promoting the 3Rs Leiden (NL): Leiden University; [ https://www.risk-hunt3r.eu/.

[22] Precision Tox -. Towards Precision Toxicology. Available from: https://precisiontox.org/.

[23] PARC - Partnership. for the Assessment of Risks from Chemicals. Available from: https://www.eu-parc.eu/.

[24] Benjamini Y, Hochberg Y (1995). Controlling the false Discovery rate: a practical and powerful Approach to multiple testing. J Roy Stat Soc: Ser B (Methodol).

[25] Wang C, Gong B, Bushel PR, Thierry-Mieg J, Thierry-Mieg D, Xu J (2014). The concordance between RNA-seq and microarray data depends on chemical treatment and transcript abundance. Nat Biotechnol.

[26] Lyu Y, Li Q (2016). A semi-parametric statistical model for integrating gene expression profiles across different platforms. BMC Bioinformatics.

[27] TECHNICAL_NOTE. <clariom-d-taqman-concordance-technical-note.pdf>. Concordance of Clariom D microarrays and TaqMan real-time PCR assays. 2018. [Internet]. Available from: https://assets.thermofisher.com/TFS-Assets%2FGSD%2FTechnical-Notes%2Fclariom-d-taqman-concordance-technical-note.pdf. Accessed 07 July 2023.

