# Supplementary material for: A comparison of the TempO-Seq and Affymetrix microarray platform using RTqPCR validation
Source: BMC Genomics. 2024 Jul 3;25:669. doi: 10.1186/s12864-024-10586-7 (PMC11223392; doi:10.1186/s12864-024-10586-7)
Supplement: Supplementary file 1 — Supplementary Material 1 [file 12864_2024_10586_MOESM1_ESM.pdf]

Project report for BioClavis service project (BCL-SP0018)

Fraunhofer – ExiTox

Overview and purpose

This report describes the results (both QC and experimental samples) and links to results files generated from the TempO-Seq assays performed in this study. The work described in this report is a study using targeted sequencing-based RNA expression analysis on purified RNA.

If you have any questions, contact BioClavis, either a member of the project team or support@bioclavis.co.uk.

Sample information and plate layouts

Samples were supplied in five 96-well plates (received 29/05/2018), containing 288 samples as below, and were analysed using the Human whole transcriptome panel v1.1 with standard attenuators.

Plate Name: A549-Vinylester-ALI

	1	2	3	4	5	6	7	8	9	10	11	12
A	Pos Control 1A	1_B1_3	1_B5_1	2_R1_1	2_B3_2	3_NEC1_1	3_B2_a_3					
B	Pos Control 1B	1_R1_1	1_B5_2	2_NEC1_1	2_B3_3	3_B2_1	3_R2_a_1					
C	Pos Control 2A	1_NEC1_1	1_B5_3	2_B2_1	2_R3_1	3_B2_2	3_NEC2_a_1					
D	Pos Control 2B	1_B4_1	1_R5_1	2_B2_2	2_NEC3_1	3_B2_3						
E	Neg Control 1	1_B4_2	1_NEC5_1	2_B2_3	3_B1_1	3_R2_1						
F	Neg Control 2	1_B4_3	2_B1_1	2_R2_1	3_B1_2	3_NEC2_1						
G	1_B1_1	1_R4_1	2_B1_2	2_NEC2_1	3_B1_3	3_B2_a_1						
H	1_B1_2	1_NEC4_1	2_B1_3	2_B3_1	3_R1_1	3_B2_a_2						

A3	1_B5_1	50ng/µl
B3	1_B5_2	50ng/µl
C3	1_B5_3	50ng/µl

Plate Name:A549-Sek-Amine-ALI

	1	2	3	4	5	6	7	8	9	10	11	12
A	Pos Control 1A	8_B1_3	8_B3_2	6_R1_1	6_B6_2	7_NEC1_1	7_B3_4	13_B2_1	13_R3_1			
B	Pos Control 1B	8_R1_1	8_B3_3	6_NEC1_1	6_B6_3	7_B2_1	7_R3_1	13_B2_2	13_NEC3_1			
C	Pos Control 2A	8_NEC1_1	8_B3_4	6_B5_1	6_R6_1	7_B2_2	7_NEC3_1	13_B2_3				
D	Pos Control 2B	8_B2_1	8_R3_1	6_B5_2	6_NEC6_1	7_B2_3	13_B1_1	13_R2_1				
E	Neg Control 1	8_B2_2	8_NEC3_1	6_B5_3	7_B1_1	7_R2_1	13_B1_2	13_NEC2_1				
F	Neg Control 2	8_B2_3	6_B1_1	6_R5_1	7_B1_2	7_NEC2_1	13_B1_3	13_B3_1				
G	8_B1_1	8_R2_1	6_B1_2	6_NEC5_1	7_B1_3	7_B3_1	13_R1_1	13_B3_2				
H	8_B1_2	8_NEC2_1	6_B1_3	6_B6_1	7_R1_1	7_B3_2	13_NEC1_1	13_B3_3				

Plate Name:A549-Sek_Aldehyde-ALI

	1	2	3	4	5	6	7	8	9	10	11	12
A	Pos Control 1A	9_B1_3	9_B3_1	10_R1_1	10_B3_2	11_NEC1_1	11_B2_3	12_B2_1	12_R3_1	22_B2_2	22_NEC3_1	
B	Pos Control 1B	9_R1_1	9_B3_2	10_NEC1_1	10_B3_3	11_B4_1	11_R2_1	12_B2_2	12_NEC3_1	22_B2_3		
C	Pos Control 2A	9_NEC1_1	9_B3_3	10_B2_1	10_R3_1	11_B4_3	11_NEC2_1	12_B2_3	22_B1_1	22_R2_1		
D	Pos Control 2B	9_B2_1	9_R3_1	10_B2_2	10_NEC3_1	11_B4_4	12_B1_2	12_R2_1	22_B1_2	22_NEC2_1		
E	Neg Control 1	9_B2_2	9_NEC3_1	10_B2_3	11_B1_1	11_R4_2	12_B1_3	12_NEC2_1	22_B1_3	22_B3_1		
F	Neg Control 2	9_B2_3	10_B1_1	10_R2_1	11_B1_2	11_NEC4_2	12_B1_4	12_B3_1	22_R1_1	22_B3_2		
G	9_B1_1	9_R2_1	10_B1_2	10_NEC2_1	11_B1_3	11_B2_1	12_R1_1	12_B3_2	22_NEC1_1	22_B3_3		
H	9_B1_2	9_NEC2_1	10_B1_3	10_B3_1	11_R1_1	11_B2_2	12_NEC1_1	12_B3_3	22_B2_1	22_R3_1		

Plate Name:A549-Acrylate-subm

	1	2	3	4	5	6	7	8	9	10	11	12
A	Pos Control 1A	14_B3_1	14_B9_1	15_B6_1	16_B3_1	16_B9_1	17_B6_2	22_B3_1	22_B9_1			
B	Pos Control 1B	14_K1_1	14_K3_1	15_K2_1	16_K1_1	16_K3_1	17_K2_1	22_K1_1	22_K3_1			
C	Pos Control 2A	14_B4_1	15_B1_1	15_B7_1	16_B4_1	17_B1_1	17_B7_1	22_B4_1				
D	Pos Control 2B	14_B5_1	15_B2_1	15_B8_1	16_B5_1	17_B2_1	17_B8_1	22_B5_1				
E	Neg Control 1	14_B6_1	15_B3_1	15_B9_1	16_B6_1	17_B3_1	17_B9_1	22_B6_1				
F	Neg Control 2	14_K2_1	15_K1_1	15_K3_1	16_K2_1	17_K1_1	17_K3_1	22_K2_1				
G	14_B1_1	14_B7_1	15_B4_1	16_B1_1	16_B7_1	17_B4_1	22_B1_1	22_B7_1				
H	14_B2_1	14_B8_1	15_B5_1	16_B2_1	16_B8_1	17_B5_1	22_B2_1	22_B8_1				

Plate Name:PCLuS-Acrylate-subm

	1	2	3	4	5	6	7	8	9	10	11	12
A	Pos Control 1A	P12_B5_D2_1_1b	P13_B4_D2_1_1b	P14_B3_D1_1_1b	P14_B5_D4_1_1b	P15_B4_D4_1_1b	P16_C1_D4_1_1b					
B	Pos Control 1B	P12_B3_D3_1_1b	P13_B5_D2_1_1b	P14_B4_D1_1_1b	P15_B3_D2_1_1b	P15_B5_D4_1_1b	P16_C2_D4_1_1b					
C	Pos Control 2A	P12_B4_D3_1_1b	P13_B3_D3_1_1b	P14_B5_D1_1_1b	P15_B4_D2_1_1b	P16_C1_D1_1_1b	P16_C3_D4_1_1b					
D	Pos Control 2B	P12_B5_D3_1_1b	P13_B4_D3_1_1b	P14_B3_D3_1_1b	P15_B5_D2_1_1b	P16_C2_D1_1_1b	D2_K1_1b					
E	Neg Control 1	P12_B3_D4_1_1b	P13_B5_D3_1_1b	P14_B4_D3_1_1b	P15_B3_D3_1_1b	P16_C3_D1_1_1b	D3_K1_1b					
F	Neg Control 2	P12_B4_D4_1_1b	P13_B3_D4_1_1b	P14_B5_D3_1_1b	P15_B4_D3_1_1b	P16_C1_D3_1_1b	D4_K1_1b					
G	P12_B3_D2_1_1b	P12_B5_D4_1_1b	P13_B4_D4_1_1b	P14_B3_D4_1_1b	P15_B5_D3_1_1b	P16_C2_D3_1_1b						
H	P12_B4_D2_1_1b	P13_B3_D2_1_1b	P13_B5_D4_1_1b	P14_B4_D4_1_1b	P15_B3_D4_1_1b	P16_C3_D3_1_1b						

Concentration of all samples between 50ng/µl and 110ng/µl

Note: Some samples from plate 3 had the same ID as samples from plate 4, these was given a suffix of either _p3 or _p4 to distinguish during data processing.

QA/QC test results

QA/QC of BioClavis' internal process control data is comprised of the following steps, for which results are shown below. This is in addition to confirming success of the sequencing summary output

file for each sequencing run to manufacturer's spec containing the following metrics: Density (K/mm²), Clusters PF (%), Reads (M), Reads PF (M), and % ≥ Q30.

Read count analysis

Metric	Value	Status	Significance
Number of mapped reads in positive RNA controls (average)	6,683,790	Pass (>10,000 mapped reads)	Sufficient number of mapped reads are required
Signal:noise ratio (total number of mapped reads in the positive controls / total number of mapped reads in negative controls)	1431:1	Pass (>20:1)	Important quality parameter for a variety of statistical analyses.
The percentage of mapped reads in positive controls	96%	Pass (>80%)	Ensures adequate yield, not a quality score as in RNA-Seq.
Average reads / probe (>20 reads)	590	Pass (>250)	A measure of 'read depth' that demonstrates the number of times expressed genes were counted.

Replicate analysis of positive RNA controls and no-sample negative controls

Process controls are run in replicate on each assay plate of samples to ensure quality metrics pass on a plate-wise level. Representative scatter plots of log_2 transformed read counts from process control replicates are shown below. Specifically, we look for high reproducibility of two different types of positive controls, and low signal in the negative control. The assay in this project generated high quality data, as demonstrated in these measurements.

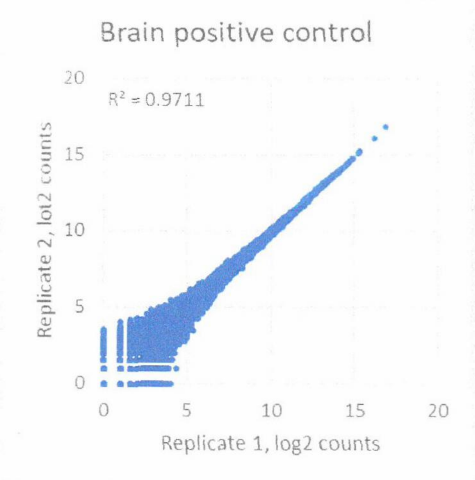

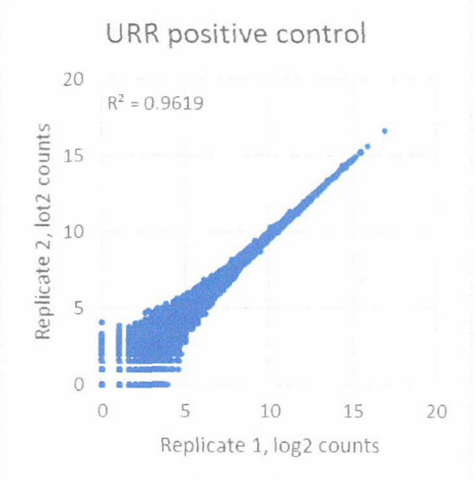

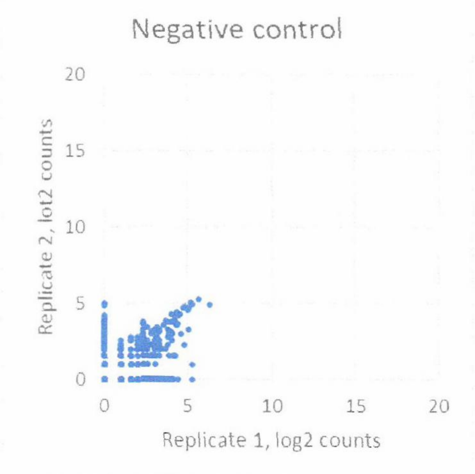

Data files

Counts per gene per sample

A data matrix of gene expression level (raw counts) with sample names as column headers and gene names as rows is the primary output of this study. The data file 'Counts per gene per sample_raw_ExiTox_BCL-SP0018.csv' will be delivered on a hard drive as well as transferred electronically.

Attenuation

Note, some commonly high expressed genes have been attenuated in this assay resulting in their counts being reduced in a linear and predictable manner. The list of these attenuated probes is included in the manifest. The result with attenuated counts are perfectly valid for fold difference calculations, since attenuation impacts all samples the same. However, if you would like a data file with these genes counts 'back calculated' to what they would have been, for example in comparing absolute counts across different platforms, we can provide these. Note, this calculation requires normalisation of the dataset.

Normalised counts per gene per sample

Normalisation is typically not necessary and as such the data provided have not been normalised.

Sequencing output

Raw sequencing data files in FASTQ format will be delivered on a hard drive.

Protocols

TempO-Seq assay protocol summary

Sequencing libraries for targeted panels were generated as described briefly below and depicted in the Figure.

In TempO-Seq, each Detector Oligo consists of a sequence complementary to an mRNA target plus a universal (i.e. same for every targeted gene) primer binding site. They anneal in immediate juxtaposition to each other on the targeted RNA template such that they can be ligated together. Ligated detector oligos are PCR-amplified using a primer set (single-plex PCR reaction, with a single primer pair for each sample) that introduces both the adaptors required for sequencing and a sample-specific barcode. The barcode sequences flank the target sequence and are inserted appropriately into the standard Illumina adaptors to permit standard dual-index sequencing of the barcodes and deconvolution of sample-specific reads from the sequencing data using the standard Illumina software. All the PCR-amplified and barcoded samples are pooled into a single library for sequencing. Sequencing reads are demultiplexed using the standard sequencing instrument software for each sample using the barcodes to give a FASTQ file for each.

Figure: Schematic of TempO-Seq assay protocol

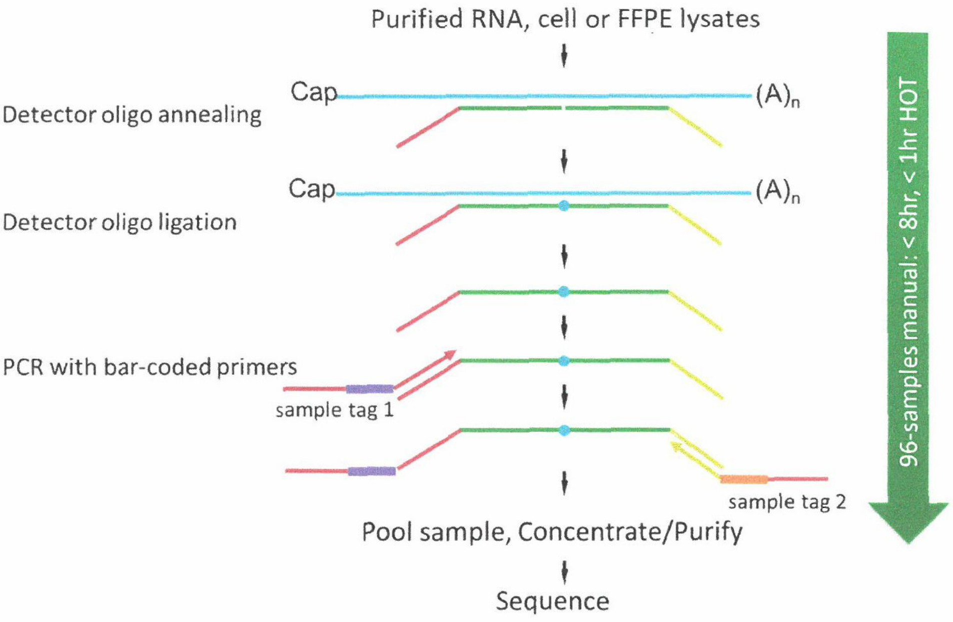

Data analysis protocol

TempO-Seq sequence files were analysed using the Tempo-SeqR software package. The input for TempO-Seq data analysis is a folder of zipped FASTQ files. Each FASTQ file contains the reads and quality scores for one sample. Each FASTQ file is aligned using the Bowtie algorithm to a pseudo-transcriptome corresponding to the gene panel used in the assay. It takes ~3 minutes to align 1 million reads using the Tempo-SeqR software. The primary output of the Tempo-SeqR software is a table of counts with each column representing a sample and each row representing a gene.

Data normalisation

Data normalisation is commonly not necessary. A variety of normalisation methods may be appropriate for various downstream analyses at your discretion, including normalisation to total reads per sample.

Probe manifest

ProbeID is composed of gene name along with a numeric identifier, separated by an underscore. Some genes may have multiple ProbeIDs representing assays against isoforms that are not captured by a single probe against the region of maximal overlap of all gene isoforms. For additional information about probe location, including target sequence and RefSeq ID, refer to the Probe Manifest file that will be delivered on a hard drive.

Return of remaining materials

If you would like the remaining RNA and libraries returned at your cost, please request shipment within 3 months unless arrangements were already made. You will need to supply a FedEx account number to charge against. We cannot guarantee that any materials are left or warrant their quality but will attempt to satisfy requests as much as possible.
