# Supplementary material for: A comparison of the TempO-Seq and Affymetrix microarray platform using RTqPCR validation
Source: BMC Genomics. 2024 Jul 3;25:669. doi: 10.1186/s12864-024-10586-7 (PMC11223392; doi:10.1186/s12864-024-10586-7)
Supplement: Supplementary file 3 — Supplementary Material 3 [file 12864_2024_10586_MOESM3_ESM.docx]

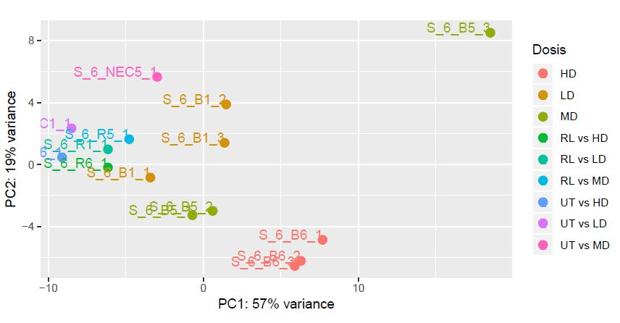


Supplementary Figure 1: Principal component analysis (PCA) of log normalized gene counts from TempO-Seq sequencing measurements. One sample labelled S_6_B5_3 in the top right corner of the plot shows a clear separation from other samples on principal component 1 (PC1) and was removed as an outlier from further analysis.


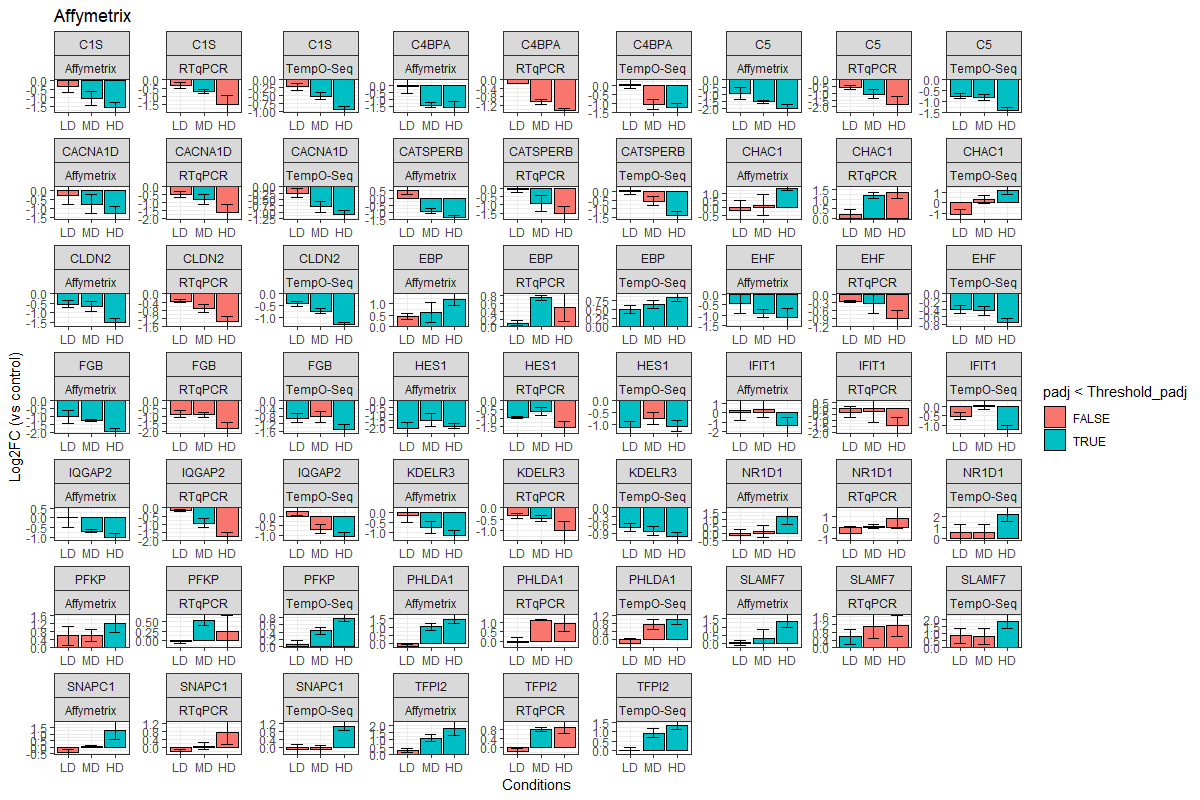


Supplementary Figure 2: The figure shows log2 transformed fold changes (log2FC) for 20 non-confirmed genes as measured by 3 different technologies (Affymetrix, RTqPCR, and TempO-Seq). Each barplot shows log2FCs for the dose levels low dose (LD), mid dose (MD), and high dose (HD). Bars are coloured turquoise when the adjusted p-value for the respective change lies under the threshold of 0.05. Dose dependent differential expression can be observed for most presented genes as well as high concordance among the platforms. For these genes differential expression was not confirmed by RTqPCR at the HD dose level.


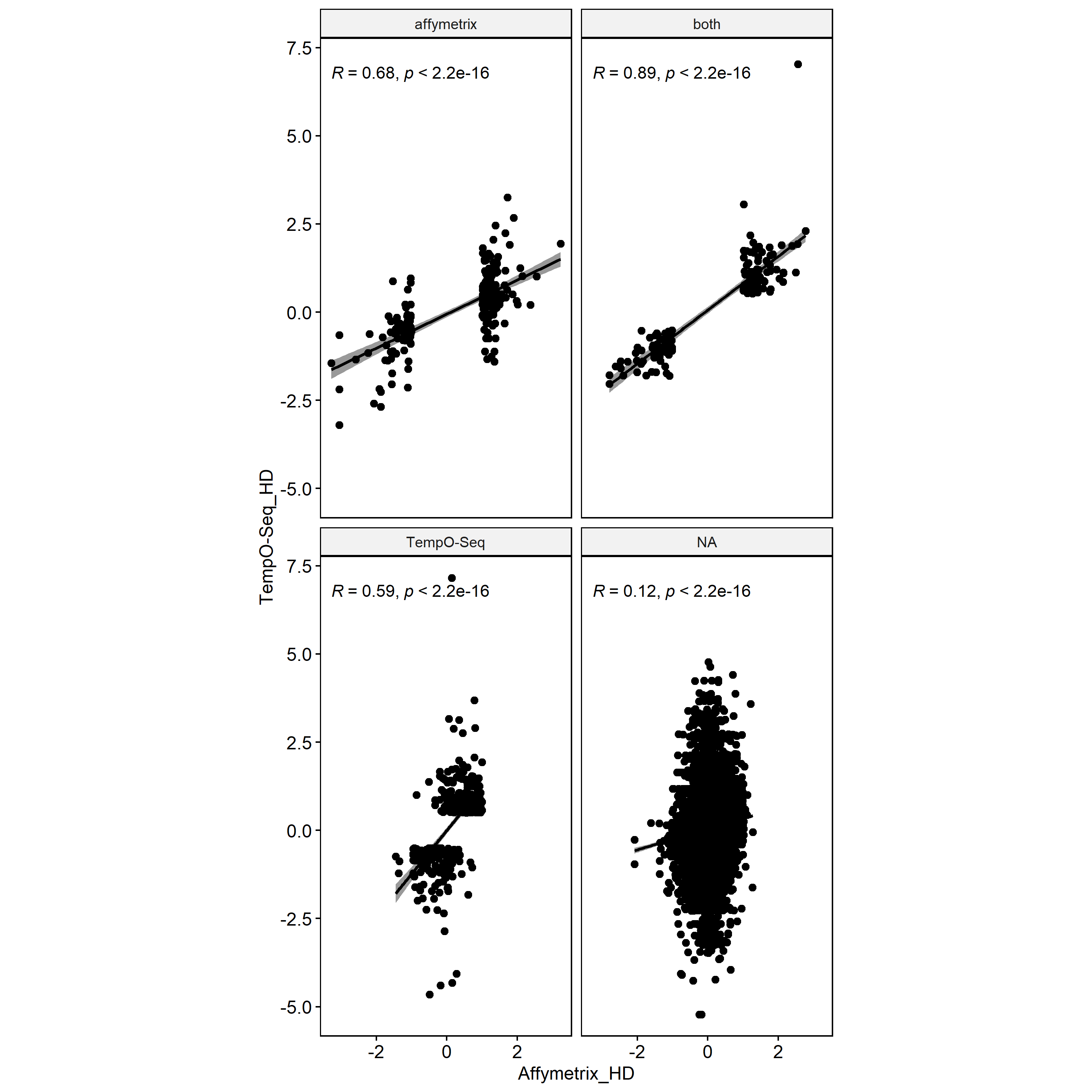


Supplementary Figure 3: The scatterplots show log 2 transformed fold changes (log2FC) for the two platforms Affymetrix and TempO-Seq. The individual panels show I) Affymetrix –differentially expressed genes (DEGs) in the Affymetrix platform, II) both – DEGs according to both platforms, III) TempO-Seq – DEGs in the TempO-Seq platform and IV) NA – genes which showed no significant differential expression in either platform. The spearman-correlation coefficient R is highest for II) genes which are determined to be DEG in both platforms.
